# Inhibition of PI3K/AKT/mTOR signaling enhances autophagy in HL-60 acute myeloid leukemia cells: An integrative bioinformatic and in vitro study

**DOI:** 10.1016/j.bbrep.2025.102220

**Published:** 2025-08-27

**Authors:** Mohammad Malekan, Armin Dozandeh-Jouybari, Nazanin Joudaki, Mehdi Ahangari, Reza Valadan, Hossein Asgarian-Omran, Saeid Taghiloo

**Affiliations:** a Student Research Committee, School of Medicine, Mazandaran University of Medical Sciences, Sari, Iran; b Department of Immunology, School of Medicine, Mazandaran University of Medical Sciences, Sari, Iran; c Molecular and Cell Biology Research Center, Mazandaran University of Medical Sciences, Sari, Iran; d Gastrointestinal Cancer Research Center, Non-Communicable Diseases Institute, Mazandaran University of Medical Sciences, Sari, Iran; e Immunogenetics Research Center, Mazandaran University of Medical Sciences, Sari, Iran

**Keywords:** Acute myeloid leukemia, PI3K/AKT/mTOR, Autophagy, Idelalisib, MK 2206, Everolimus

## Abstract

**Background:**

Acute myeloid leukemia (AML) involves uncontrolled proliferation of myeloid progenitor cells and carries a poor prognosis. The PI3K/AKT/mTOR pathway plays a key role in AML pathogenesis by regulating cancer cell proliferation and survival. This study investigates the effects of inhibiting the PI3K/AKT/mTOR pathway on autophagy in AML cell lines, aiming to support targeted therapy development that modulates autophagy.

**Methods:**

Gene expression and prognostic significance of PI3K/AKT/mTOR and autophagy-related genes in AML were evaluated using Enricher, GEPIA2, and Human Protein Atlas databases. HL-60 cells were treated with Idelalisib, MK-2206, and Everolimus, selective PI3K, AKT, and mTOR inhibitors, either individually or in combination. Autophagy gene expression (*Beclin-1, LC3-II, Atg5, ATG7*) was assessed by Real-time PCR.

**Result:**

Bioinformatic analysis revealed that autophagy genes are associated with PI3K/AKT/mTOR pathway in AML. We observed that HL-60 AML cell lines treated with PI3K/AKT/mTOR inhibitors exhibited significant enhancement in the expression of key autophagy-related genes, including *Beclin-1*, *LC3-II*, *ATG5*, and *ATG7*, particularly with combination treatment.

**Conclusion:**

PI3K/AKT/mTOR inhibitors significantly induce autophagy-related gene expression in AML cells. These findings support combining such inhibitors with autophagy modulators as a potential strategy to improve AML treatment outcomes.

## Highlights

• PI3K/AKT/mTOR inhibition enhances autophagy in AML cells.
• Combination therapy upregulates Beclin-1, LC3-II, ATG5, and ATG7.
• Autophagy genes linked to PI3K/AKT/mTOR signaling in AML.
• Targeted PI3K/AKT/mTOR inhibition may improve AML therapy.
• Bioinformatics confirms autophagy-pathway association in AML.

## 1 Introduction

Acute myeloid leukemia (AML) is the most common adult acute leukemia with heterogeneous characteristics. It is also the second most common cause of leukemia in children. AML is defined as an uncontrolled proliferation of myeloid progenitor cells that leads to bone marrow failure through a complex process [1,2]. In addition to bone marrow, malignant cells can infiltrate into extramedullary tissues and be found in peripheral blood [3]. AML results in neutropenia, anemia, and thrombocytopenia in patients. The main presentations of the disease include fatigue, dizziness, shortness of breath during normal activities, bruising, bleeding, fever, recurrent infections, and impaired wound healing [4]. The prognosis of AML remains unsatisfactory, especially in elderly patients, as approximately two-thirds of them experience relapse following polychemotherapy, highlighting an unmet need for new therapeutic options [5,6].

Recent advances in understanding the underlying cellular and molecular features of AML and providing targeted therapies open new hope for therapeutic progress in patients [7]. The phosphoinositide 3 kinase/Akt/mammalian (or mechanistic) target of rapamycin (PI3K/AKT/mTOR) pathway plays a crucial role in both normal and malignant hematopoiesis. In AML, this pathway is pivotal in regulating cell proliferation, differentiation, survival, and apoptosis. The dysregulation of this pathway is commonly associated with the pathogenesis of AML, leading to the uncontrolled growth and resistance to apoptosis [8]. Additionally, the activation of the PI3K/AKT/mTOR pathway through the detection of associated genes and protein expression was linked to lower overall survival (OS) and disease-free survival (DFS) in 50–80 % of AML patients [6,9].

Targeting the PI3K/AKT/mTOR signaling pathway appears to be essential for AML patients. Inhibitors that target this pathway have demonstrated promising results in preclinical studies, although their clinical efficacy in AML has been considered disappointing based on the outcomes of multiple clinical trials [10]. Studies have suggested that investigating the inhibition of the PI3K/AKT/mTOR pathway in AML patients through various settings and designs may significantly improve outcomes. This can be achieved with the use of new-generation inhibitors, combination therapies with inhibitors of other activated signaling pathways, traditional forms of chemotherapy, and dual mTOR/PI3K inhibitors or catalytic mTOR inhibitors [2]. Furthermore, autophagy, an essential cellular degradation process, has garnered significant attention in the context of PI3K/AKT/mTOR inhibition [11].

Autophagy is an intracellular recycling process of macromolecules and damaged organelles through lysosomal digestion [12]. This process exerts multiple effects, including serving as a cellular survival and stress-relief mechanism, thereby maintaining homeostasis. Autophagy-related genes and proteins include *ATGs* such as *ATG5* and *ATG7*, *Beclin-1*, *LC3*, *P62/SQSTM1*, and *Bcl-2* [13]. Furthermore, it has been established that the PI3K/AKT/mTOR pathway plays an essential role in regulating autophagy [14]. Autophagy has been considered a double-edged sword in cancer such as AML, as it can act as both a cancer cell suppressor and a survival mechanism concurrently [15].

Therefore, understanding how to modulate autophagy in AML is crucial for developing therapeutic strategies that effectively leverage its efficacy in targeted therapies such as PI3K/AKT/mTOR inhibitors. This requires a nuanced approach, balancing the tumor-suppressive properties of autophagy with the risk of supporting cancer cell survival and resistance mechanisms. This study aims to investigate the impact of PI3K/AKT/mTOR pathway inhibition on autophagy in AML cell lines, to identify potential therapeutic interventions that can enhance the treatment of AML through targeted modulation of autophagy.

## 2 Materials and methods

### 2.1 Bioinformatics analysis

The Enrichr tool (https://maayanlab.cloud/Enrichr/) was used to investigate the significance of the PI3K/AKT/mTOR signaling pathway and autophagy-related genes across various cancer types and cellular processes [16,17]. The GEPIA2 database (https://gepia2.cancer-pku.cn/) was employed to analyze the expression patterns of PIK3CA, AKT1, and mTOR in AML patients compared to normal samples, using data from The Cancer Genome Atlas (TCGA-LAML) and Genotype-Tissue Expression (GTEx) datasets. Specifically, expression analysis included 173 AML samples and 70 normal samples. Survival analysis was conducted using GEPIA2 to assess the relationship between PIK3CA, AKT1, and mTOR expression and overall survival (OS) in AML patients. For survival analysis, a subset of 106 AML patients with available survival data from the TCGA-LAML dataset was used, divided into high and low expression groups (n = 53 each) based on the median expression level of each gene. The Human Protein Atlas database (https://www.proteinatlas.org/) was utilized to evaluate the expression levels of PIK3CA, AKT1, and mTOR across common cell lines.

### 2.2 Reagents

The compounds Idelalisib, MK-2206, and Everolimus were procured from Cayman Chemical Company in Michigan, USA, functioning as a PI3K, Akt, and mTOR inhibitors, respectively. They were dissolved in dimethyl sulfoxide (DMSO) and stored in frozen aliquots. This research received ethical approval from the Ethical Committee of Mazandaran University of Medical Sciences (IR.MAZUMS.REC.1403.045).

### 2.3 Cultured cell lines and treatment groups

The HL-60 cell line, representative of AML, was acquired from the Pasteur Institute in Tehran, Iran. Cells were cultured in RPMI-1640 medium (Biowest, Nuaille, France), supplemented with 10 % fetal bovine serum (FBS) from Biowest, along with 100 U/mL of penicillin and 100 μg/mL of streptomycin (Biowest). The culture was maintained in an environment with 5 % carbon dioxide at 37 °C. The optimal concentrations, or half-maximal inhibitory concentration (IC_50_) values, for all pharmacological agents were assessed using the MTT assay [18]. The HL-60 cell line was then exposed to the optimal concentration of signaling inhibitors for a period of 48 h. The research involved three separate monotherapy groups, namely Idelalisib, MK-2206, and Everolimus, in addition to four groups that utilized combination therapies, which included Idelalisib and MK-2206, Idelalisib and Everolimus, MK-2206 and Everolimus, and a combination of all three: Idelalisib, MK-2206, and Everolimus.

### 2.4 RNA isolation and real-time polymerase chain reaction

Total RNA extraction was carried out utilizing the Denazist Asia kit (Mashhad, Iran) in accordance with the manufacturer's guidelines. The process of reverse transcription aimed at producing complementary DNA (cDNA) from total RNA was accomplished using the Yekta-Tajhiz cDNA synthesis kit (Tehran, Iran), incorporating Molony murine leukemia virus reverse transcriptase alongside random hexamers. For assessing the relative expression levels of *Beclin-1*, a protein encoded by the *BECN1* gene in humans, along with *LC3-II*, ATG5, and *ATG7*, Real-time polymerase chain reaction (PCR) was conducted employing the amplicon (Copenhagen, Denmark) SYBER Green PCR master mix reagents utilizing a StepOne Real-time PCR system (Applied Biosystems, Foster City, CA, USA). AllelID software facilitated the primer design, which is detailed in Table 1. Preliminary trials were executed to ensure the efficiency of the primers during the reactions, leading to the construction of standard curves. For data normalization, *β-actin* was employed as a housekeeping gene [18,19], and the Pfaffl method was implemented to derive relative expression values. Each experiment was performed in triplicate.

**Table 1:** Primer sequences used for Real-Time PCR.

Gene	Sequences (5′-3′)	Product Size (bp)
***Beclin-1***	**F:** ATCAGGAGGAAGCTCAGTAT	104 bp
	**R:** GGCATAACGCATCTGGTT	

***LC3-II***	**F:** AAGAGTAGAAGATGTCCGA	125 bp
	**R:** GGTCAGGTACAAGGAACT	

***ATG-5***	**F:** CACAAGCAACTCTGGATG	136 bp
	**R:** CAGTCGTTGTCTGATATATTCTA	

***ATG-7***	**F:** AGGAGATTCAACCAGAGAC	170 bp
	**R:** GAGGCTCATTCATCCGAT	

***β-actin***	**F:** CCTTCCTGGGCATGGAGTCCT	174 bp
	**R:** TGGGTGCCAGGGCAGTGAT	

F: Forward primer, R: Reverse primer.

### 2.5 Statistical analysis

The analysis of the results was conducted utilizing GraphPad Prism 9 software. Data were expressed as the mean ± standard deviation (SD). The Kolmogorov-Smirnov test was employed to assess the normality of the data distribution. A one-way analysis of variance (ANOVA) was performed, accompanied by the Dunnett test for multiple comparisons between the treatment groups and the control group [20]. Additionally, Spearman's rank correlation was applied to determine the correlation coefficients between continuous variables with normal distributions. P-values less than 0.05 were considered indicative of statistically significant differences.

## 3 Results

### 3.1 Enrichment analysis of PI3K/AKT/mTOR pathway and autophagy-related genes

**Fig. 1:**
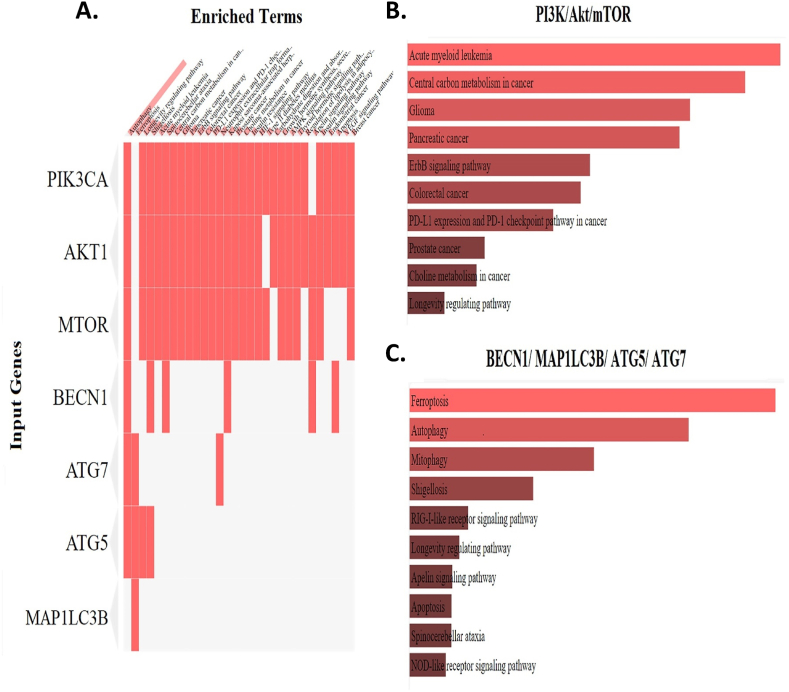
Enrichment analysis of PI3K/AKT/mTOR pathway and autophagy-related genes across different cancers and various cellular processes using the Enricher database (https://maayanlab.cloud/Enrichr/). A) Enriched terms of *PIK3CA*, *AKT1*, *mTOR*, *BECN1*, *ATG7*, *ATG5*, and *MAP1LC3B*. B) The PI3K/AKT/mTOR signaling pathway in different situation. C) Isolated analysis of *BECN1*, *ATG7*, *ATG5*, and *MAP1LC3B* genes across different cellular processes.

The enrichment analysis was conducted to exhibit the activity of the PI3K/AKT/mTOR signaling pathway and autophagy genes, including *BECN1*, *ATG7*, *ATG5*, and *MAP1LC3B*. This was done across different cancers and various cellular processes, both unfavorable and favorable, using the Enricher website (Fig. 1). This data revealed a significant enrichment of *PIK3CA*, *AKT1*, *mTOR*, *BECN1*, *ATG7*, and *ATG5* in autophagy. In AML, the analysis showed that *PIK3CA*, *AKT1*, and *mTOR* were enriched, while *BECN1*, *MAP1LC3B*, *ATG7*, and *ATG5* were silent. This suggests that upregulation of the PI3K/AKT/mTOR pathway is associated with decreased autophagy in AML (Fig. 1A). Among the cancers analyzed, AML exhibited the highest activity of the PI3K/AKT/mTOR pathway (Fig. 1B). Isolated analysis of *BECN1*, *ATG7*, *ATG5*, and *MAP1LC3B* genes across different cellular processes showed the highest enrichment in ferroptosis, autophagy, and mitophagy, respectively (Fig. 1C).

### 3.2 Expression and survival analysis of *PIK3CA*, *AKT1*, and *mTOR* in AML

**Fig. 2:**
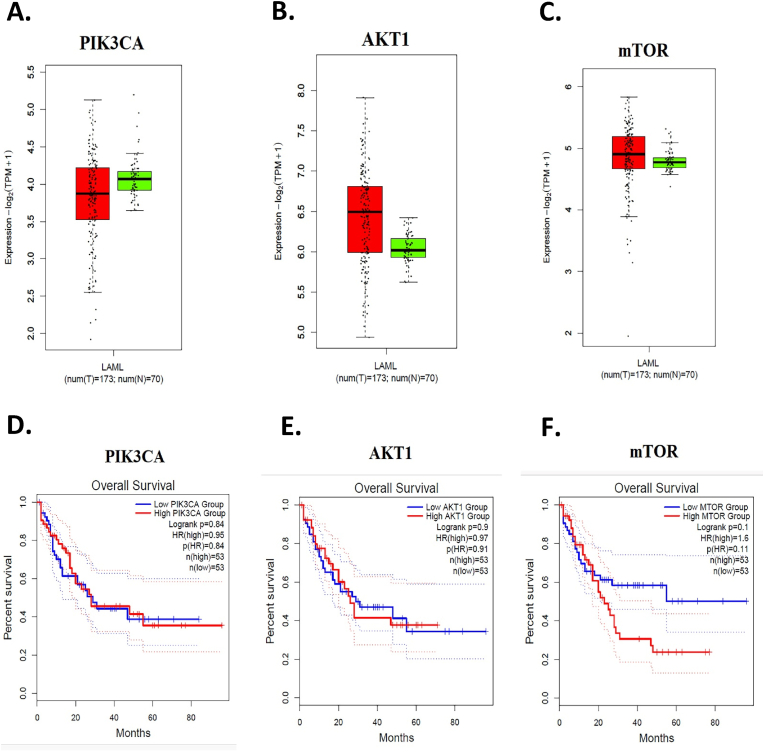
Expression and survival analysis of *PIK3CA*, *AKT1*, and *mTOR* in AML patients using the GEPIA2 database (https://gepia2.cancer-pku.cn/). **A-C)** The expression pattern of *PIK3CA*, *AKT1*, and *mTOR* in 173 AML patients compared with 70 normal samples. Red: AML samples, Green: normal samples. **D-F)** The association of *PIK3CA*, *AKT1*, and *mTOR* with overall survival in 106 AML patients (53 patients with high expression and 53 patients with low expression). Blue: low expression group, Red: high expression group.

We analyzed the expression pattern of *PIK3CA*, *AKT1*, and *mTOR* in 173 AML patients compared with 70 normal samples using the GEPIA2 database (Fig. 2). The expression of *PIK3CA* was no different in AML compared to the normal samples (Fig. 2A). However, the expression of *AKT1* and *mTOR* was higher in the AML group (Fig. 2B and C). The association of *PIK3CA*, *AKT1*, and *mTOR* with the overall survival of 106 AML patients (53 patients with high expression and 53 patients with low expression) is depicted in Fig. 2D–F through survival analysis using the GEPIA2 database. The analysis indicates that the overall survival rates of AML patients with high and low expression of *PIK3CA* and *AKT1* showed no difference (Fig. 2D and E, Hazard Ratio (HR) = 0.95, p = 0.84 and HR = 0.97, p = 0.91, respectively). Nevertheless, the results showed that the high *mTOR* expression is associated with reduced overall survival compared to the low *mTOR* group, indicating that high *mTOR* expression in AML patients increases the hazard of death compared to low expression (Fig. 2F, HR = 1.6, p = 0.11).

### 3.3 Comparative analysis of *PIK3CA*, *AKT1*, and *mTOR* gene expression in different common cell lines

**Fig. 3:**
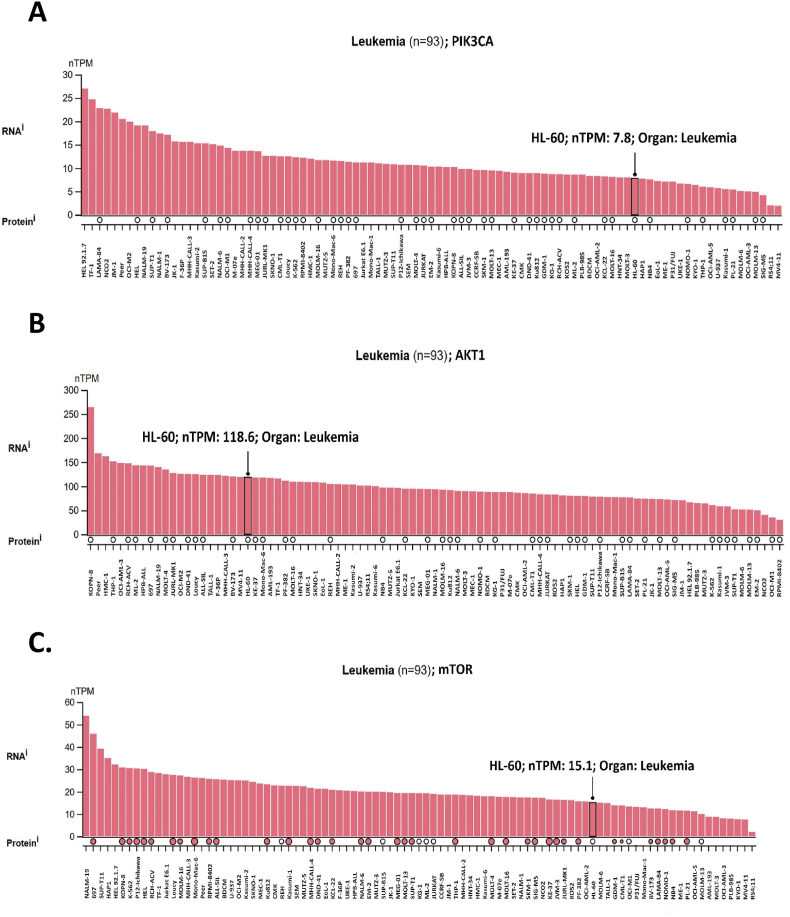
The expression levels of *PIK3CA*, *AKT1*, and *mTOR* in different leukemia cell lines using the Human Protein Atlas database (https://www.proteinatlas.org/). A) *PIK3CA*. B) *AKT1*. C) *mTOR*.

We analyzed the expression of the *PIK3CA*, *AKT1*, and *mTOR* genes across various leukemia cell lines using the Human Protein Atlas database, with a specific focus on the HL-60 cell line, which was employed in our study (Fig. 3). The results indicated high mRNA expression of *PIK3CA, AKT1, and mTOR* in HL-60 cell line, with a measured level of 7.8, 118.6 and 15.1 nTPM (normalized Transcripts Per Million, Fig. 3A and B, and C), respectively.

### 3.4 Evaluation of autophagy-related gene expression under PI3K/AKT/mTOR pathway inhibition in HL-60 cell line

**Fig. 4:**
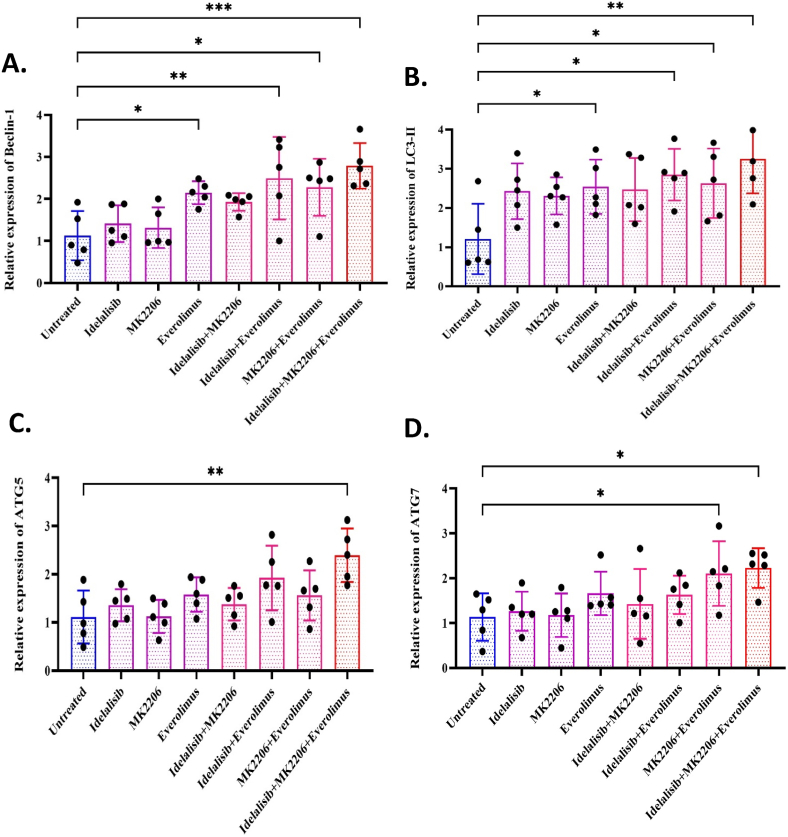
Effects of PI3K/AKT/mTOR inhibitors on the expression of autophagy-related gene expression. HL-60 cells were cultured in the absence or presence of Idelalisib, MK2206, and Everolimus for 48 h either in single or combined treatment. After that, total RNA was extracted, and cDNA was synthesized. Real-time PCR was performed with specific primers for *Beclin-1*, *LC3-II*, *ATG5*, and *ATG7*. A) Relative mRNA transcript levels of *Beclin-1*. B) Relative mRNA transcript levels of LC3-II. C) Relative mRNA transcript levels of *ATG5*. D) Relative mRNA transcript levels of *ATG7*. Gene expression results are represented as mean ± SD of the Pfaffl method after normalization with β-actin as an internal control. One-way ANOVA with Dunnett post hoc test was used for analyses. ∗p < 0.05; ∗∗p < 0.01; ∗∗∗p < 0 0.001; ∗∗∗∗p < 0.0001.

We measured the mRNA expression levels of autophagy-related genes including *Beclin-1*, *LC3-II*, *ATG5*, and *ATG7* under various treatment conditions using Idelalisib, MK2206, and Everolimus as PI3K/AKT/mTOR inhibitors in the HL-60 AML cell line (Fig. 4). As expected for a commonly used housekeeping gene, *β-actin* expression remained stable across treatment conditions in HL-60 cells, consistent with previous reports of its use in this cell line [21]. Our results exhibited an increase in the expression of *Beclin-1* after single or combined treatment with inhibitors compared to the untreated group. The upregulation of *Beclin-1* expression was significant in single treatment with Everolimus (p-value <0.05) and in the co-treatment groups: Idelalisib with Everolimus (p-value <0.01), MK2206 with Everolimus (p-value <0.05), and the combination of Idelalisib, MK2206, and Everolimus (p-value <0.001), compared to the untreated group (Fig. 4A). Similarly, *LC3-II* expression was significantly elevated in single treatment with Everolimus (p-value <0.05) and in the co-treatment groups: Idelalisib with Everolimus (p-value <0.05), MK2206 with Everolimus (p-value <0.05), and the combination of Idelalisib, MK2206, and Everolimus (p-value <0.01), compared to the untreated group (Fig. 4B). The *ATG5* expression is exhibited in Fig. 4C, where a significant increase is noted in the co-treatment group with Idelalisib, MK2206, and Everolimus compared to the untreated group (p-value <0.01). However, in the other treated groups, no significant changes were observed compared to the untreated group. As shown in Fig. 4D, the expression of *ATG7* was significantly increased in the co-treatment group with MK2206 and Everolimus (p-value <0.05) and in the combination of Idelalisib, MK2206, and Everolimus (p-value <0.05) compared to the untreated group. These findings indicate that PI3K/AKT/mTOR inhibitors, particularly the combined treatment with Idelalisib, MK2206, and Everolimus, significantly enhance the expression of key autophagy-related genes, suggesting a potential synergistic effect in promoting autophagy.

### 3.5 Correlation analysis of autophagy-related gene expression and total apoptosis after PI3K/AKT/mTOR pathway inhibition in HL-60 cell line

**Fig. 5:**
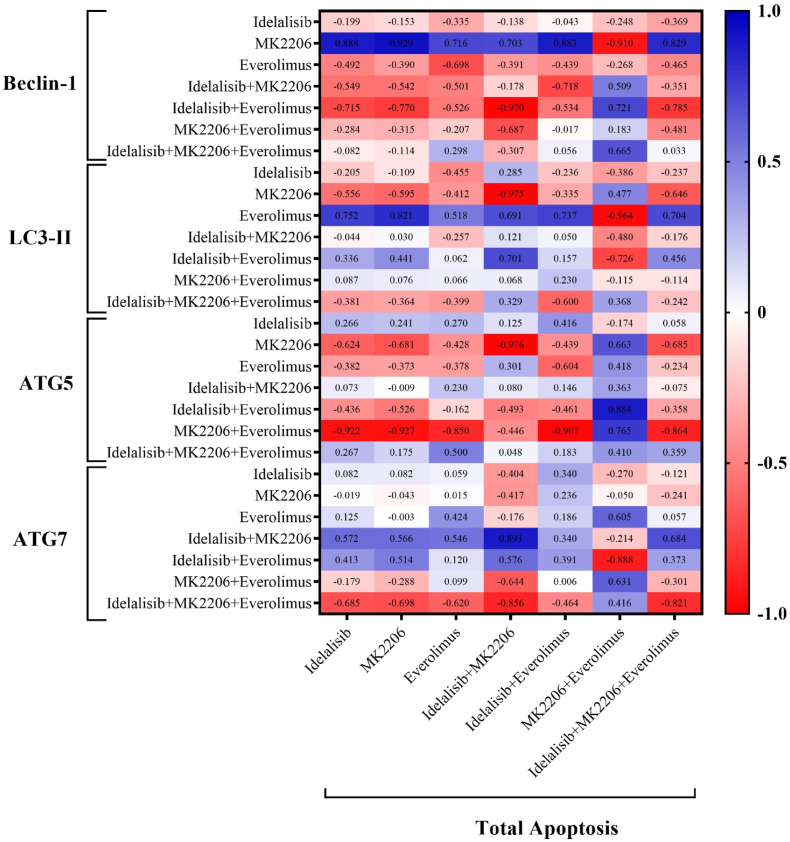
The heat map of correlation analysis based on *Beclin-1*, *LC3-II*, *ATG5*, and *ATG7* expression and total apoptosis following treatment with PI3K/AKT/mTOR pathway inhibitors. Spearman's rank correlation was applied to determine the correlation coefficients.

We have shown in our previous study that the total apoptosis of HL-60 cells after treatment with Idelalisib, MK-2206, and Everolimus alone or in combination was increased when compared to the untreated group [18]. To find any correlations between the autophagy-related gene expression, including *Beclin-1*, *LC3-II*, *ATG5*, and *ATG7*, and their relationship with total apoptosis, the current results were analyzed with our previous data. These results highlight the significant inverse relationship between autophagy-related protein expression and total apoptosis, particularly under combination treatment with Idelalisib, MK2206, and Everolimus (Fig. 5). The heat map shown in Fig. 5 displays the correlation coefficients between various treatment groups in more detail.

## 4 Discussion

Our study demonstrates that inhibition of the PI3K/AKT/mTOR signaling pathway in HL-60 AML cells significantly upregulates key autophagy-related genes, including *Beclin-1*, *LC3-II*, *ATG5*, and *ATG7*. This core finding indicates that PI3K/AKT/mTOR inhibitors may induce autophagy in AML cells. These observations were consistent across experimental data and bioinformatic enrichment analyses, which revealed that autophagy-related genes are functionally linked to PI3K/AKT/mTOR activity in AML.

The PI3K/AKT/mTOR pathway is a central regulator of cell growth, survival, and metabolism. Targeting the PI3K/AKT/mTOR signaling pathways has garnered significant interest in the treatment of cancer, including AML, resulting in the development of various small molecule agents [6]. Despite the addition of targeted agents, including PI3K/AKT/mTOR pathway inhibitors and novel chemotherapy regimens, AML relapse remains a common issue [22,23].

Understanding the complex processes involved in AML progression and the mechanisms leading to drug resistance is necessary to sensitize cancer cells to chemotherapeutic agents. In our work, we employed three agents with distinct inhibitory targets within this pathway. We used Idelalisib, as a PI3K inhibitor [18], MK-2206 as a pan-Akt inhibitor [24], and Everolimus as an mTORC1 inhibitor [25,26]. Combined treatment with these inhibitors amplified autophagy-related gene expression, suggesting possible synergistic effects.Preclinical studies on the efficacy of PI3K/AKT/mTOR inhibitors in AML cell lines and primary AML cells have yielded promising results [9]. However, clinical studies in this area have been limited and the results have been regarded as disappointing by clinicians [6].

The impact of these therapeutic strategies is influenced by multiple factors, including cancer type, disease stage, tumor microenvironment, and the underlying genetic context [27,28]. Recent exome-wide analyses have identified genetic variants significantly associated with AML drug combination responses [29].

Studies have proposed several solutions regarding these disappointing outcomes [2,3]. One of them is incorporating agents that can modulate or block autophagy in cancer cells, which is a crucial process that are activated in tumor cell lines [30]. This approach is particularly important because of the various synergistic effects of PI3K/AKT/mTOR inhibitors, including autophagy induction, apoptosis induction, and alterations in metabolism [19,31]. However, the debate surrounding the role of autophagy in cancer is divided into two opposing effects: tumor suppression and tumor progression [12,32].

On the one hand, autophagy prevents tumor initiation, particularly in the early stages of cancer progression, through multiple mechanisms [33]. On the other hand, the deletion of autophagy-related genes has been shown to promote tumor formation, such as the deletion of *ATG7* in liver tumors [34] and the deletion of *Beclin-1* in breast cancer [35]. Furthermore, in the later stages, autophagy can activate defensive mechanisms within tumor cells, leading to their survival and alleviating stress, hypoxia, DNA damage, and metabolic stress [36]. This process supports tumor metabolism and growth, facilitating tumor development. Consequently, it fosters tumorigenesis and contributes to resistance against therapeutic agents [13,36].

Autophagy modulation through PI3K/AKT/mTOR inhibition has been documented in multiple AML models. Kumar et al. demonstrated that Fascaplysin induces autophagy, evidenced by the upregulation of *LC3-II*, *ATG7*, and *Beclin-1*. Additionally, an increase in pro-apoptotic events, such as PARP-1 cleavage and caspase activation, occurs through the inhibition of the PI3K/AKT/mTOR signaling pathway in HL-60 cell lines [37]. Zhang et al. indicated that Tanshinone IIA (Tan IIA) induces autophagy through the upregulation of *LC3-II*, *ATG5*, and *Beclin-1* in the U937 AML cell line via the downregulation of the PI3K/Akt pathway [38]. Pan et al. conducted a study similar to that of Zhang et al. but on NB4 cell lines. They observed that Tanshinone IIa induces autophagy and apoptosis in acute promyelocytic leukemia NB4 cells through the downregulation of the PI3K/AKT/mTOR pathway. This is evidenced by the upregulation of *LC3B* and the activation of apoptotic markers like cleaved-caspase 9, cleaved-caspase 3, and cleaved-PARP-1. This study further reveals that Tan IIa-induced autophagy can be reversed by the autophagy inhibitor Baf-A1, highlighting its role in NB4 cell apoptosis [39].

Of note, our correlation analysis reveals a significant inverse relationship between autophagy-related protein expression and total apoptosis. This effect is particularly evident in cells treated with the combination of Idelalisib, MK2206, and Everolimus. Recent studies by Lin et al. and Zhu et al. have shown a similar negative correlation between autophagy and apoptosis through the alteration of the PI3K/AKT/mTOR pathway [40,41]. Effects of these complex processes on AML drug resistance were also evaluated. Chen et al. illustrated that T-complex protein 1 (TCP1) enhances drug resistance in AML by inhibiting autophagy and apoptosis triggered by Adriamycin through the activation of the AKT/mTOR signaling pathway. This is shown by the increased TCP1 expression in AML patients, which is linked to lower complete response rates and reduced overall survival. Additionally, the interaction of TCP1 with AKT and mTOR in HL-60 and K562 cell lines further supports these findings [42].

While our study provides important insights into the regulation of autophagy by PI3K/AKT/mTOR inhibitors in AML cells, several limitations should be acknowledged. First, the experiments were conducted primarily in the HL-60 cell line, and responses may differ across other AML models or patient-derived samples. Second, all analyses were performed in vitro, and the effects observed may not fully reflect the complex tumor microenvironment in vivo. Third, although we observed correlations between autophagy-related gene expression and apoptosis, causal relationships and the precise mechanisms underlying drug resistance were not fully explored. Fourth, functional validation of autophagy at the protein level and through autophagic flux assays was not included, limiting mechanistic confirmation. Finally, bioinformatic analyses relied on publicly available datasets, which may have inherent biases and may not perfectly recapitulate experimental conditions.

**Fig. 6:**
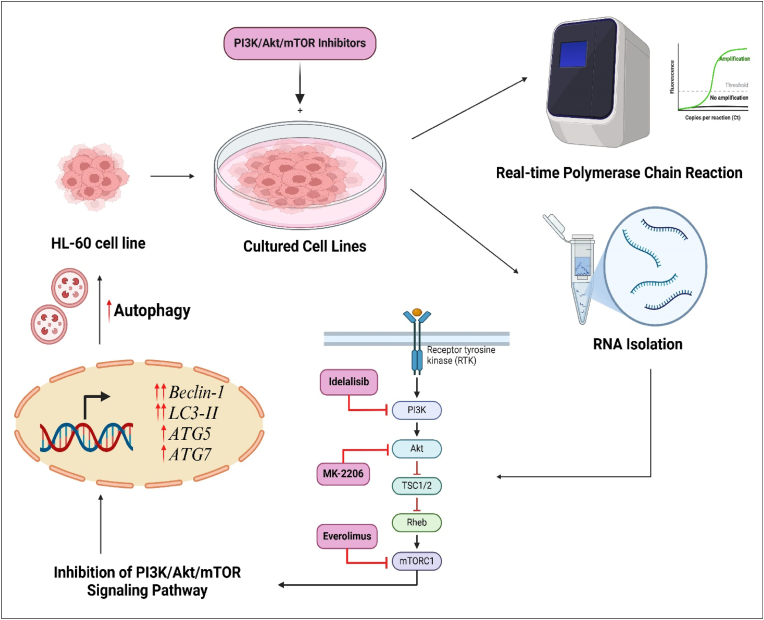
HL-60 cells were cultured and treated with selective inhibitors of PI3K (Idelalisib), AKT (MK-2206), and mTOR (Everolimus). RNA was isolated from treated cells and analyzed using real-time PCR to quantify the expression of key autophagy-related genes, including *Beclin-1, LC3-II, ATG5*, and *ATG7*. The inhibition of the PI3K/AKT/mTOR signaling pathway resulted in upregulation of these genes, indicating enhanced autophagy in AML cells.

In conclusion, our study uncovers a critical link between autophagy and the PI3K/AKT/mTOR signaling pathway in AML cells, demonstrating that combined treatment with PI3K/AKT/mTOR inhibitors leads to significant upregulation of autophagy-related genes such as *Beclin-1, LC3-II, ATG5*, and *ATG7* (Fig. 6). This suggests that these inhibitors may induce autophagy as a potential mechanism leading to drug resistance. The findings underscore the potential therapeutic value of combining PI3K/AKT/mTOR inhibitors with autophagy modulators to overcome drug resistance and improve treatment outcomes, particularly in future clinical trials.

## Author contributions

**Mohammad Malekan**: Conceptualization, Methodology, Investigation, Writing – original draft, Data curation, Validation. **Armin Dozandeh-Jouybari:** Investigation, Data curation, Formal analysis. **Nazanin Joudaki**: Investigation, Data curation, Visualization. **Mehdi Ahangari**: Methodology, Resources, Formal analysis. **Reza Valadan**: Methodology, Investigation, Software. **Hossein Asgarian-Omran**: Supervision, Project administration, Writing – review & editing. **Saeid Taghiloo**: Conceptualization, Methodology, Formal analysis, Writing – review & editing, Supervision, Project administration.

## Data availability

Data will be available upon reasonable request from the corresponding author.

## Funding

This study was supported by 10.13039/501100004160Mazandaran University of Medical Sciences (Project number: 21801).

## Declaration of competing interest

The authors declare no actual or potential conflict of interest.

## References

[1] Zhang N., Shen M.Y., Meng Q.L., Sun H.P., Fan F.Y., Yi H. (2024). FAT1 inhibits AML autophagy and proliferation via downregulating ATG4B expression. Biochim. Biophys. Acta, Gen. Subj..

[2] Herschbein L., Liesveld J.L. (2018). Dueling for dual inhibition: means to enhance effectiveness of PI3K/Akt/mTOR inhibitors in AML. Blood Rev..

[3] Nepstad I., Hatfield K.J., Grønningsæter I.S., Reikvam H. (2020). The PI3K-Akt-mTOR signaling pathway in human acute myeloid leukemia (AML) cells. Int. J. Mol. Sci..

[4] Blackburn L.M., Bender S., Brown S. (2019). Acute leukemia: diagnosis and treatment. Semin. Oncol. Nurs..

[5] Yilmaz M., Wang F., Loghavi S., Bueso-Ramos C., Gumbs C., Little L. (2019). Late relapse in acute myeloid leukemia (AML): clonal evolution or therapy-related leukemia?. Blood Cancer J..

[6] Darici S., Alkhaldi H., Horne G., Jørgensen H.G., Marmiroli S., Huang X. (2020). Targeting PI3K/Akt/mTOR in AML: rationale and clinical evidence. J. Clin. Med..

[7] Wang X., Zhong L., Dan W., Chu X., Luo X., Liu C. (2023). MiR-454-3p promotes apoptosis and autophagy of AML cells by targeting ZEB2 and regulating AKT/mTOR pathway. Hematology (Amsterdam, Netherlands).

[8] Evangelisti C., Evangelisti C., Bressanin D., Buontempo F., Chiarini F., Lonetti A. (2013). Targeting phosphatidylinositol 3-kinase signaling in acute myelogenous leukemia. Expert Opin. Ther. Targets.

[9] Bertacchini J., Heidari N., Mediani L., Capitani S., Shahjahani M., Ahmadzadeh A. (2015). Targeting PI3K/AKT/mTOR network for treatment of leukemia. Cell. Mol. Life Sci. : CMLS.

[10] Fransecky L., Mochmann L.H., Baldus C.D. (2015). Outlook on PI3K/AKT/mTOR inhibition in acute leukemia. Molecular and cellular therapies.

[11] Xu Z., Han X., Ou D., Liu T., Li Z., Jiang G. (2020). Targeting PI3K/AKT/mTOR-mediated autophagy for tumor therapy. Appl. Microbiol. Biotechnol..

[12] Debnath J., Gammoh N., Ryan K.M. (2023). Autophagy and autophagy-related pathways in cancer. Nat. Rev. Mol. Cell Biol..

[13] Li X., He S., Ma B. (2020). Autophagy and autophagy-related proteins in cancer. Mol. Cancer.

[14] Heras-Sandoval D., Pérez-Rojas J.M., Hernández-Damián J., Pedraza-Chaverri J. (2014). The role of PI3K/AKT/mTOR pathway in the modulation of autophagy and the clearance of protein aggregates in neurodegeneration. Cell. Signal..

[15] Joffre C., Ducau C., Poillet-Perez L., Courdy C., Mansat-De Mas V. (2021). Autophagy a close relative of AML biology. Biology.

[16] Chen E.Y., Tan C.M., Kou Y., Duan Q., Wang Z., Meirelles G.V. (2013). Enrichr: interactive and collaborative HTML5 gene list enrichment analysis tool. BMC Bioinf..

[17] Kuleshov M.V., Jones M.R., Rouillard A.D., Fernandez N.F., Duan Q., Wang Z. (2016). Enrichr: a comprehensive gene set enrichment analysis web server 2016 update. Nucleic acids research.

[18] Taghiloo S., Norozi S., Asgarian-Omran H. (2022). The effects of PI3K/Akt/mTOR signaling pathway inhibitors on the expression of immune checkpoint ligands in acute myeloid leukemia cell line. Iran. J. Allergy Asthma Immunol..

[19] Ranjbar A., Soltanshahi M., Taghiloo S., Asgarian-Omran H. (2023). Glucose metabolism in acute myeloid leukemia cell line is regulated via combinational PI3K/AKT/mTOR pathway inhibitors. Iran. J. Pharm. Res. (IJPR) : IJPR.

[20] Dunnett C.W. (1955). A multiple comparison procedure for comparing several treatments with a control. J. Am. Stat. Assoc..

[21] Barber R.D., Harmer D.W., Coleman R.A., Clark B.J. (2005). GAPDH as a housekeeping gene: analysis of GAPDH mRNA expression in a panel of 72 human tissues. Physiol. Genom..

[22] Dykstra K.M., Fay H.R.S., Massey A.C., Yang N., Johnson M., Portwood S. (2021). Inhibiting autophagy targets human leukemic stem cells and hypoxic AML blasts by disrupting mitochondrial homeostasis. Blood Adv..

[23] Bower H., Andersson T.M., Björkholm M., Dickman P.W., Lambert P.C., Derolf Å.R. (2016). Continued improvement in survival of acute myeloid leukemia patients: an application of the loss in expectation of life. Blood Cancer J..

[24] Sandhöfer N., Metzeler K.H., Rothenberg M., Herold T., Tiedt S., Groiß V. (2015). Dual PI3K/mTOR inhibition shows antileukemic activity in MLL-rearranged acute myeloid leukemia. Leukemia.

[25] Yang J., Ikezoe T., Nishioka C., Ni L., Koeffler H.P., Yokoyama A. (2010). Inhibition of mTORC1 by RAD001 (everolimus) potentiates the effects of 1,25-dihydroxyvitamin D(3) to induce growth arrest and differentiation of AML cells in vitro and in vivo. Exp. Hematol..

[26] Soltanshahi M., Taghiloo S., Asgarian-Omran H. (2022). Expression modulation of immune checkpoint molecules by ibrutinib and everolimus through STAT3 in MCF-7 breast cancer cells. Iran. J. Pharm. Res. (IJPR) : IJPR.

[27] Kumar A., Singh U.K., Chaudhary A. (2015). Targeting autophagy to overcome drug resistance in cancer therapy. Future Med. Chem..

[28] Hanahan D. (2022). Hallmarks of cancer: new dimensions. Cancer Discov..

[29] Giri A.K., Lin J., Kyriakidis K., Tripathi G., Almusa H. (2025). Exome-wide association study reveals 7 functional variants associated with ex-vivo drug response in acute myeloid leukemia patients. BMC Med. Genom..

[30] Zhao Y., Zou Z., Sun D., Li Y., Sinha S.C., Yu L. (2021). GLIPR2 is a negative regulator of autophagy and the BECN1-ATG14-containing phosphatidylinositol 3-kinase complex. Autophagy.

[31] Li X.Q., Cheng X.J., Wu J., Wu K.F., Liu T. (2024). Targeted inhibition of the PI3K/AKT/mTOR pathway by (+)-anthrabenzoxocinone induces cell cycle arrest, apoptosis, and autophagy in non-small cell lung cancer. Cell. Mol. Biol. Lett..

[32] Amaravadi R.K., Kimmelman A.C., Debnath J. (2019). Targeting autophagy in cancer: recent advances and future directions. Cancer Discov..

[33] Sun K., Deng W., Zhang S., Cai N., Jiao S., Song J. (2013). Paradoxical roles of autophagy in different stages of tumorigenesis: protector for normal or cancer cells. Cell Biosci..

[34] Takamura A., Komatsu M., Hara T., Sakamoto A., Kishi C., Waguri S. (2011). Autophagy-deficient mice develop multiple liver tumors. Genes & development.

[35] Yue Z., Jin S., Yang C., Levine A.J., Heintz N. (2003). Beclin 1, an autophagy gene essential for early embryonic development, is a haploinsufficient tumor suppressor. Proc. Natl. Acad. Sci. U. S. A..

[36] Wu W.K., Coffelt S.B., Cho C.H., Wang X.J., Lee C.W., Chan F.K. (2012). The autophagic paradox in cancer therapy. Oncogene.

[37] Kumar S., Guru S.K., Pathania A.S., Manda S., Kumar A., Bharate S.B. (2015). Fascaplysin induces caspase mediated crosstalk between apoptosis and autophagy through the inhibition of PI3K/AKT/mTOR signaling cascade in human leukemia HL-60 cells. J. Cell. Biochem..

[38] Zhang Y., Geng Y., He J., Wu D., Zhang T., Xue L. (2019). Tanshinone IIA induces apoptosis and autophagy in acute monocytic leukemia via downregulation of PI3K/Akt pathway. Am. J. Tourism Res..

[39] Pan Y., Chen L., Li R., Liu Y., Nan M., Hou L. (2021). Tanshinone IIa induces autophagy and apoptosis via PI3K/Akt/mTOR Axis in acute promyelocytic leukemia NB4 cells. Evid. base Compl. Alternative Med. : eCAM.

[40] Lin C.Y., Chen Y.H., Huang Y.C. (2023). Hesperetin induces autophagy and delayed apoptosis by modulating the AMPK/Akt/mTOR pathway in human leukemia cells in vitro. Curr. Issues Mol. Biol..

[41] Zhu T., Zhang H., Li S., Wu K., Yin Y., Zhang X. (2022). Detoxified pneumolysin derivative ΔA146Ply inhibits autophagy and induces apoptosis in acute myeloid leukemia cells by activating mTOR signaling. Exp. Mol. Med..

[42] Chen X., Chen X., Huang Y., Lin J., Wu Y., Chen Y. (2021). TCP1 increases drug resistance in acute myeloid leukemia by suppressing autophagy via activating AKT/mTOR signaling. Cell Death Dis..

